# Effects of adiponectin, plasma D-dimer, inflammation and tumor markers on clinical characteristics and prognosis of patients with ovarian cancer

**DOI:** 10.5937/jomb0-26452

**Published:** 2022-02-02

**Authors:** Hui Li, Lulu Sun, Lili Chen, Zhihui Kang, Guorong Hao, Fenglou Bai

**Affiliations:** 1 The Fourth Hospital of Shijiazhuang (Obstetrics and Gynaecology Hospital), Department of Gynecology, Shijiazhuang City, Hebei Province, China; 2 The Fourth Hospital of Shijiazhuang (Obstetrics and Gynaecology Hospital), Department of Obstetrics, Shijiazhuang City, Hebei Province, China

**Keywords:** adiponectin, plasma D-dimer, inflammatory factors, tumour markers, ovarian cancer, adiponektin, D-dimer u plazmi, inflamatorni faktori, tumorski markeri, rak jajnika

## Abstract

**Background:**

To investigate the effects of adiponectin (ADPN), plasma D-dimer (D-D), inflammation, and tumour markers on clinical characteristics and prognosis of patients with ovarian cancer.

**Methods:**

A total of 80 patients with ovarian cancer treated in our hospital from April 2017 to November 2019 were enrolled as study subjects and evenly divided into an observation group (patients with ovarian cancer) and a control group (patients with the benign ovarian tumour) based on the results of the postoperative pathological biopsy. The levels of ADPN, plasma D-D, inflammatory factors, and serum tumour markers carbohydrate antigen 125 (CA125), human epididymis protein 4 (HE4), and risk of ovarian malignancy algorithm (ROMA) were compared between the two groups. The diagnostic value of serum tumour markers CA125, HE4, and ROMA in ovarian cancer was explored. The correlations of ROMA changes with the changes in the levels of ADPN, plasma D-D, high-sensitivity C-reactive protein (hs-CRP), CA125, and HE4 were analysed. Additionally, the related risk factors affecting the development of ovarian cancer were subjected to univariate and multivariate logistic regression analyses.

**Results:**

In comparison with the control group, the observation group exhibited a lowered ADPN level (p<0.05), notably raised levels of plasma D-D, inflammatory factors hs-CRP and interleukin-6 (IL-6) and serum tumour markers CA125 and HE4 and an evidently increased ROMA (p<0.05). Besides, the detection of serum ROMA showed the highest specificity and sensitivity and low false-positive rate and false-negative rate. The changes of ROMA were positively correlated with the changes in the levels of plasma D-D, hs-CRP, CA125, and HE4 (p<0.05) and negatively associated with the changes in ADPN level (p<0.05). The results of the univariate analysis showed that abnormal ADPN, D-D, hs-CRP, IL-6, CA125, and HE4 levels were related to risk factors affecting the development of ovarian cancer. It was found through multivariate logistic regression analysis that decreased ADPN level and increased D-D, hsCRP, IL-6, CA125, and HE4 levels were independent risk factors affecting the development of ovarian cancer.

**Conclusions:**

In the case of ovarian cancer, the ADPN level declines, while the levels of plasma D-D, inflammatory factors, and serum tumour markers CA125, HE4, and ROMA rise obviously. Besides, the ROMA level displays a positive relation to the content of CA125, HE4, plasma D-D, and inflammatory factors and a negative association with the ADPN level.

## Abstract (sr)

**Uvod:**

Cilj ove studije bio je da ispita efekte adiponektina (ADPN), D-dimera u plazmi (D-D), upala i tumorskih markera na kliničke karakteristike i prognozu kod pacijentkinja sa rakom jajnika.

**Metode:**

U studiju je uključeno ukupno 80 pacijentkinja sa rakom jajnika lečenih u našoj bolnici od aprila 2017. do novembra 2019. godine. Pacijentkinje su ravnomerno podeljene u grupu za posmatranje (pacijentkinje sa rakom jajnika) i kontrolnu grupu (pacijentkinje sa benignim tumorom jajnika) na osnovu rezultata postoperativne biopsije. Između dve grupe upoređivani su nivoi ADPN, D-D u plazmi, inflamatorni faktori i serumski tumorski markeri ugljenohidratni antigen 125 (CA125), humani protein epididimisa 4 (HE4) i algoritam rizika za malignitet jajnika (ROMA). Istražena je dijagnostička vrednost tumorskih markera u serumu CA125, HE4 i ROMA kod karcinoma jajnika. Analizirane su korelacije promena ROMA sa prome nama nivoa ADPN, D-D u plazmi, C-reaktivnog proteina visoke osetljivosti (hs-CRP), CA125 i HE4. Pored toga, povezani faktori rizika koji utiču na razvoj raka jajnika podvrgnuti su univarijantnim i multivarijantnim logističkim regresionim analizama.

**Rezultati:**

U poređenju sa kontrolnom grupom, posmatrana grupa je imala sniženi nivo ADPN (p < 0,05), značajno povišene nivoe D-D u plazmi, inflamatornog faktora hs-CRP i interleukina-6 (IL-6) i serumskih tumorskih markera CA125 i HE4 i jasno uvećan ROMA (p < 0,05). Pored toga, otkrivanje serumskog ROMA pokazalo je najveću specifičnost i osetljivost i nisku stopu lažno pozitivnih i lažno negativnih rezultata. Promene ROMA su u pozitivnoj korelaciji sa promenama nivoa D-D u plazmi, hs-CRP, CA125 i HE4 (p < 0,05), a negativno su povezane sa promenama u nivou ADPN (p <0,05). Rezultati univarijantne analize pokazali su da su abnormalni nivoi ADPN, D-D, hs-CRP, IL-6, CA125 i HE4 povezani sa faktorima rizika koji utiču na razvoj karcinoma jajnika. Kroz multivarijantnu logističku regresionu analizu utvrđeno je da su smanjeni nivo ADPNa i povećani nivoi D-D, hs-CRP, IL-6, CA125 i HE4 bili nezavisni faktori rizika koji utiču na razvoj karcinoma jajnika.

**Zaključak:**

U slučaju karcinoma jajnika, nivo ADPN opada, dok nivoi D-D u plazmi, inflamatorni faktori i tumorski markeri u serumu CA125, HE4 i ROMA očigledno rastu. Pored toga, nivo ROMA pokazuje pozitivnu vezu sa sadržajem CA125, HE4, D-D u plazmi i inflamatornim faktorima, a negativnu povezanost sa nivoom ADPN.

## Introduction

Patients with ovarian cancer tend to be at the middle or advanced stage when definitely diagnosed, and some of them even lost the opportunities for surgery [1]. Ovarian cancer, the most common gynaecological malignancy, is characterized by an insidious onset, inconspicuous early symptoms, challenging diagnosis, and a poor prognosis, so it has a high fatality rate [2]. The knowledge, as well as diagnosis and treatment methods for ovarian tumours, have been significantly improved as the living standards of people and economic level elevate, and medical technology has developed rapidly in China. However, the fatality and disability rates of ovarian cancer remain high [3].

Studies have manifested that the 5-year survival rate of patients with early ovarian cancer is more than 90% after regular treatment [4], and that of those with middle or advanced ovarian cancer, especially those who have lost opportunities for surgical treatment, is less than 20% [5]. For this reason, improving the survival rate and quality of life of patients with ovarian cancer is the focus and hotspot in current studies. Early diagnosis and early treatment of ovarian cancer are particularly important. However, most patients seek medical advice only after complications occur since they have no specific symptoms and signs in the early stage [6]. In addition, the specificity and sensitivity of tumour markers applied in the diagnosis of ovarian cancer in clinical practice need to be improved [7]. A study denoted that patients with malignant tumours, including those with ovarian cancer, have significant changes in the levels of adiponectin (ADPN), plasma D-dimer (D-D), and related inflammatory factors [8]. In this study, there-fore, the effects of ADPN, plasma D-D, inflammation, and tumour markers on the prognosis of ovarian cancer were explored. It is now reported as follows.

## Materials and Methods

### General data

A total of 80 patients with ovarian cancer treated with surgery in our hospital from April 2017 to November 2019 were enrolled as study subjects. After surgery, these patients were definitely diagnosed based on the results of the pathological biopsy. All subjects signed enrolment consent. This study was approved by the Ethics Committee of our hospital. The diagnosis and treatment were conducted as per the fourth edition of the *Guidelines for the Diagnosis and Treatment of Ovarian Malignancies* (2015). Before surgery, a tentative diagnosis was made combined with the patient's clinical symptoms, imaging examination findings, and relevant biochemical auxiliary examinations. Then, surgical treatment was carried out. After the surgery, patients underwent regular chemoradiotherapy and biologic therapy. All patients enrolled had complete clinical data and were followed up regularly. Exclusion criteria were: patients with mental illness, those undergoing neoadjuvant chemotherapy before enrolment, those with unclear diagnosis, those lost to follow-up, those with respiratory failure, long-term administration of glucocorticoids, those with systemic immune system diseases or severe liver and kidney dysfunction, those who had not signed the enrolment consent, those with malignant tumours in other parts, those with a KPS score less than 60, or those with tumour cachexia. The enrolled patients were evenly divided into an observation group (patients with ovarian cancer) and a control group (patients with the benign ovarian tumour)based on the results of the postoperative pathologicalbiopsy. In the observation group, patients were aged 19–60 years old with (49.9 ± 2.7) on average, theduration of gynaecological symptoms was 1–6 monthwith a mean of (2.8 ± 0.5) months, the tumour diameter was 1–15 cm with an average of (8.1 ± 2.1) cm,and 21 had ascites. In the control group, the age was18–60 years old with (50.0 ± 2.6) on average, theduration of gynaecological symptoms was 1–6 monthwith a mean of (2.9 ± 0.6) months, the tumour diameter was 1–15 cm with an average of (8.0 ± 2.0) cm,and 20 had ascites. There were no statistically significant differences in the age, duration of gynaecological symptoms, tumour diameter, and the ratio ofascites, as well as the levels of ADPN, plasma D-D,inflammation, and tumour markers at the time ofenrolment, between the two groups (p > 0.05).

### Methods

All patients enrolled were treated with surgery and diagnosed by postoperative pathological biopsy. Before surgery, fasting elbow venous blood was collected from patients in the morning for examinations of ADPN, plasma D-D, inflammatory factors [high-sensitivity C-reactive protein (hs-CRP) and interleukin-6 (IL-6)] and tumor markers [carbohydrate antigen 125 (CA125), human epididymis protein 4 (HE4) and risk of ovarian malignancy algorithm (ROMA)]. Besides, the above-related factors affecting the incidence of ovarian cancer were analysed in all patients enrolled to determine the correlations of serum biochemical indicators with the changes in the ROMA level.

### Observation indexes

The levels of ADPN, plasma D-D, inflammatory factors, and serum tumour markers CA125, HE4, and ROMA were compared between the two groups. The diagnostic value of serum tumour markers CA125, HE4, and ROMA in ovarian cancer was explored. The correlations of the changes of ROMA with the changes in the levels of ADPN, plasma D-D, hs-CRP, CA125, and HE4 were analysed. Additionally, the related risk factors affecting the development of ovarian cancer were subjected to univariate and multivariate logistic regression analyses.

### Assessment criteria

Hs-CRP (normal value: 10 mg/L) was detected via immunoturbidimetry, and IL-6 (normal value: 0.37 - 0.46 ng/L), D-D (normal value: <200 mg/L), ADPN (normal value: 3.62 - 13.06 mg/mL), CA125 (normal value: 40 mg/L), HE4 (normal value: 70 pmol/L) and ROMA (normal value: 0 - 11.4%) were examined through enzyme-linked immunosorbent assay (ELISA).

### Statistical processing

SPSS 20.0 was used for statistical processing. Measurement data were expressed as mean±standard deviation (x̄±s). The *t*-test was used to compare the mean between two groups, and the χ^2^ test was adopted for comparison of the ratio between two groups. The correlations of ROMA level with the levels of ADPN, plasma D-D, hs-CRP, CA125, and HE4 were analysed through the Pearson method, and univariate and multivariate logistic regression analyses were performed on the related risk factors affecting the development of ovarian cancer. P < 0.05 suggested that the difference was statistically significant.

## Results

### Comparisons of the levels of ADPN, plasma D-D, and inflammatory factors between the two groups

Compared with those in the control group, the level of ADPN declined (p < 0.05), while the levels of plasma D-D and inflammatory factors his-CRP and IL-6 rose significantly in the observation group (p < 0.05) (Table 1).

**Table 1:** Comparisons of ADPN, plasma D-D and inflammatory factor levels between the two groups (x̄±s)

	ADPN<br>(mg/mL)	D-D<br>(mg/L)	Hs-CRP<br>(mL/L)	IL-6<br>(ng/L)
Observation	1.1 ± 0.2	362.3 ± 21.4	16.3 ± 2.8	0.68 ± 0.05
Control	8.6 ± 1.1	158.6 ± 8.3	8.3 ± 0.6	0.41 ± 0.02
*t*	42.426	56.128	17.669	31.710
*p*	0.000	0.000	0.000	0.000

### Comparisons of serum tumour marker levels between the two groups

The levels of CA125, HE4, and ROMA were remarkably higher in the observation group than those in the control group (p < 0.05) (Table 2).

**Table 2:** Comparisons of CA125, HE4 and ROMA levelsbetween the two groups (x̄±s)

	CA125<br>(40 mg/L)	HE4<br>(pmol/L)	ROMA<br>(%)
Observation	85.6 ± 10.5	106.8 ± 2.5	23.6 ± 1.5
Normal group	18.9 ± 2.3	45.6 ± 1.2	8.9 ± 0.6
* t*	39.245	139.578	57.547
* p*	0.000	0.000	0.000

### Diagnostic value of serum CA125, HE4, and ROMA in ovarian cancer

The detection of serum ROMA showed the highest specificity and sensitivity and low false-positive rate and false-negative rate.

### Correlation analysis of ROMA level with ADPN, plasma D-D, hs-CRP and serum CA125 and HE4 levels

**Figure 1:**
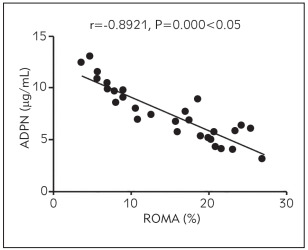
Correlation between ROMA level and ADPN level

**Figure 2:**
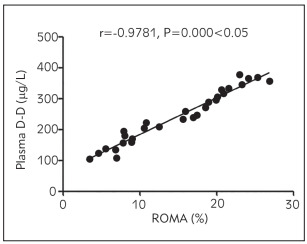
Association between ROMA level and plasma D-D level

**Figure 3:**
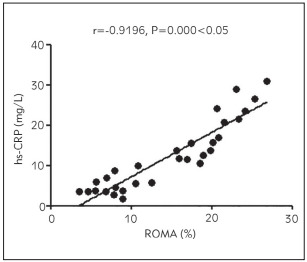
Relationship between ROMA level and hs-CRP level

**Figure 4:**
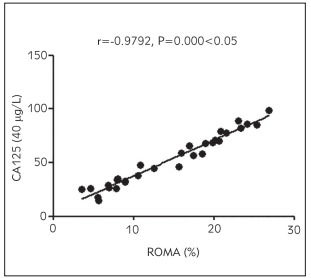
Correlation between ROMA level and serum CA125 level

**Figure 5:**
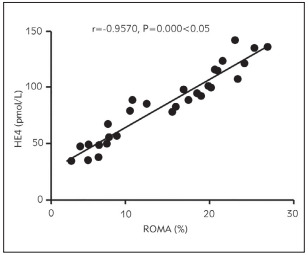
Relation between ROMA level and serum HE4 level

The ROMA level was positively associated with plasma D-D, hs-CRP, and serum CA125 and HE4 levels (p < 0.05) and negatively related to the ADPN level (p<0.05) (Figure 1, Figure 2, Figure 3, Figure 4, Figure 5) (Table 3, Table 4).

**Table 3:** Diagnostic value of serum CA125, HE4, andROMA in ovarian cancer

	Specificity	Sensitivity	False-positive rate	False-negative rate
CA125	69.8	67.3	15.6	30.8
HE4	67.8	57.8	32.1	42.1
ROMA	81.2	83.3	18.9	32.8

**Table 4:** Correlation analysis of ROMA level with ADPN,plasma D-D, hs-CRP and serum CA125 and HE4 levels

	*r *	*p *
ADPN	-0.8921	0.000
Plasma D-D	0.9781	0.000
Hs-CRP level	0.9196	0.000
CA125	0.9792	0.000
HE4	0.9570	0.000

### Univariate analysis of related risk factors affecting the incidence of ovarian cancer

It was revealed through univariate analysis that abnormal ADPN, D-D, hs-CRP, IL-6, CA125, and HE4 levels were related risk factors affecting the incidence of ovarian cancer (Table 5).

**Table 5:** Univariate analysis of related risk factors affectingthe incidence of ovarian cancer

Item		Ovarian cancer	Benign ovarian	χ2	*p*
ADPN level	Normal	9	27	19.090	0.000
	Abnormal	31	13		
D-D level	Normal	7	31	26.125	0.000
	Abnormal	33	11		
Hs-CRP level	Normal	7	26	18.620	0.000
	Abnormal	33	14		
IL-6 level	Normal	13	29	12.832	0.000
	Abnormal	27	11		
CA125 level	Normal	8	25	6.994	0.008
	Abnormal	32	15		
HE4 level	Normal	11	25	9.899	0.002
	Abnormal	29	15		

### Multivariate logistic regression analysis of related risk factors affecting the incidence of ovarian cancer

The multivariate logistic regression analysis results showed that lowered ADPN level and raised D-D, hs-CRP, IL-6, CA125, and HE4 levels were independent risk factors affecting the incidence of ovariancancer (Table 6).

**Table 6:** Multivariate logistic regression analysis of related risk factors affecting the incidence of ovarian cancer

Item	β	SE	W	OR	p	95% CI
Decreased ADPN level	1.145	0.390	11.139	5.211	0.009	0.539–2.478
Increased D-D level	1.923	0.542	4.464	2.361	0.021	1.593–9.242
Increased hs-CRP level	1.714	0.603	17.11	5.526	0.008	3.106–9.834
Increased IL-6 level	1.966	0.454	18.781	7.135	0.000	2.933–17.358
Increased CA125 level	1.605	0.382	17.717	4.972	0.000	2.354–10.490
Increased HE4 level	1.049	0.440	5.646	2.853	0.017	1.201–6.773

## Discussion

As the most common gynaecological malignant tumour in the clinic, ovarian cancer ranks first in terms of mortality rate among various gynaecological tumours [9]. As to its treatment, surgical treatment combined with postoperative adjuvant chemoradiotherapy and biological therapy is the main approach. For patients at the early stage receiving early intervention, the 5-year survival rate is over 90%, while with the progression of the disease, the 5-year survival rate is less than 20% in patients at the middle or advance stage loss the opportunities for surgery. Besides, it is prone to relapse after treatment, leading to a poor prognosis. Therefore, the early diagnosis of ovarian cancer is very important [10]. Currently, most scholars believe that many factors cause ovarian cancer, and the major ones are environmental factors, genetic factors, and lifestyle habits [11]. Besides, the prognosis of patients is affected by such factors as the clinical stage after onset, tumour histopathological type, and differentiation degree, surgical treatment, and postoperative psychological status [12]. Hence, strength ening the monitoring of patients with ovarian cancer and making early diagnosis and early treatment is of great significance for improving patients' prognosis and quality of life.

As medical technology continuously advances, the prognosis of patients with ovarian cancer has been improved significantly, especially for those with early diagnosis and treatment. However, the prognosis of patients in the middle or advanced stage remains poor. As a result, effective early diagnosis is very important for ameliorating the prognosis and quality of life and prolonging patients' survival time with ovarian cancer. The detection of tumour markers has been widely adopted in the prediction of various tumour-related diseases. The malignant tumour cells in patients with ovarian cancer will secrete and release related protein antigens during proliferation, increasing the level of responsive tumour markers. However, tumour markers with high specificity and sensitivity have not been detected yet.

In this study, the correlations of ADPN, plasma D-D, inflammatory factors, and tumour markers with the prognosis of patients were analysed. The results uncovered that compared with those in the control group, the level of ADPN declined, while the levels of plasma D-D and inflammatory factors hs-CRP and IL-6 rose significantly in the observation group, suggesting that patients with malignant ovarian tumour have a lowered ADPN level and increased levels of D-D and inflammatory factor levels. Also, the levels of serum tumour markers CA125, HE4, and ROMA were compared between the two groups. It was found that the levels of CA125, HE4, and ROMA were remarkably higher in the observation group than those in the control group, implying that patients with ovarian cancer exhibit obviously raised levels of serum CA125 and HE4 levels and an abnormal ROMA level. Moreover, the diagnostic value of serum CA125, HE4, and ROMA in ovarian cancer was explored, and it was discovered that the detection of serum ROMA showed the highest specificity and sensitivity and low false-positive rate and false-negative rate. This indicates that ROMA detection is an effective way for early comprehensive diagnosis of ovarian cancer. Furthermore, the ROMA level correlations with ADPN, plasma D-D, his-CRP, and serum CA125 and HE4 levels were analysed. The results revealed that the ROMA level was positively associated with plasma D-D, hs-CRP, and serum CA125 and HE4 levels and negatively related to ADPN level, suggesting that with the increase in ROMA, ADPN, plasma D-D, hs-CRP and serum CA125 and HE4 levels are raised, whereas ADPN level is lowered. Lastly, the results of univariate and multivariate logistic regression analyses showed that abnormal ADPN, D-D, hs-CRP, IL-6, CA125, and HE4 levels were related risk factors affecting the development of ovarian cancer, and lowered ADPN level and raised D-D, hs-CRP, IL-6, CA125, and HE4 levels were independent risk factors affecting the development of ovarian cancer.

A study demonstrated that ROMA is an ideal predictor for the risk of ovarian cancer in patients with ovarian cancer [13]. Compared with single detection and combined detection of CA125, HE4, CA724, and CEA levels, ROMA detection is more ideal and more valuable in the identification of benign and malignant gynaecological mass-related diseases in the pelvic cavity. It may be related to a larger AUC of ROMA prediction, which effectively avoids the subjective interpretation of different detection methods and different testers on the results to observably improve the diagnostic specificity and sensitivity to ovarian cancer [14]. Another study reported that the inflammatory response in vivo is negatively associated with anti-tumour immune function in patients with malignant tumours [15]. In the case of malignant tumours, the inflammatory response in vivo is significantly enhanced, leading to disorderedproliferation of tumour cells and evasion from immune surveillance, and the self-renewal, migration, metastasis, and normal apoptosis of normal cells are suppressed in the body, changing the microenvironment of tissue blood vessels and cell matrix, thereby inducing the growth of tumour cells [16].

Fat metabolism disorders and adipocyte dysfunction in tumour tissues will lead to overtly decreased ADPN level [17], and at the same time, activate the AMP-activated protein kinase (AMPK) pathway, further resulting in abnormal transduction of ADPN production signals [18]. Moreover, in malignant tumours, significant insulin resistance may occur in the body, leading to abnormal transduction of plasma insulin signals related to the occurrence of malignant tumours and abnormal energy metabolism of central sensors and cells, thus inducing malignant tumours. Finally, the research on the tumour marker CA125 revealed that CA125, which is regarded as a tumour marker related to ovarian cancer by some scholars, is generally expressed on the surface of mesothelial tissues such as the endometrium and the epithelium of the reproductive tract [19]. In the case of carcinomatosis of ovarian cells, serum CA125 is significantly increased, which can be detected in patients with malignant tumours like pancreatic cancer and breast cancer, so it only acts as a non-specific tumour marker for ovarian cancer in clinical application. HE4 is mainly secreted by ovarian cancer cells and serve as an indicator for the diagnosis of early ovarian cancer in some studies. Besides, HE4 is only secreted in early ovarian cancer cells, so the specificity and sensitivity of HE4 detection to ovarian cancer in various stages need to be improved [20].

In conclusion, patients with ovarian cancer have a reduced ADPN level and distinctly elevated levels of plasma D-D, inflammatory factors, and serum tumour markers CA125, HE4 and ROMA. Besides, the ROMA level has a positive relation to the content of CA125, HE4, plasma D-D and inflammatory factors, and a negative association with ADPN level.

## Dodatak

### Conflict of interest statement

The authors reported no conflict of interest regarding the publication of this article.

## References

[1] Albers C E, Ranjit E, Sapra A, Bhandari P, Wasey W (2020). Clinician Beware, Giant Ovarian Cysts are Elusive and Rare. Cureus.

[2] Choi J, Park C S, Seong M K, Seol H, Kim J S, Park I S, Noh W C, Kim H S (2019). Predicting the Benefit of Adjuvant Aromatase Inhibitor Therapy in Postmenopausal Breast Cancer Patients with Phosphorylated S6K1 Expression Status. J Breast Cancer.

[3] Ostmeyer J, Lucas E, Christley S, Lea J, Monson N, Tiro J, Cowell L G (2020). Biophysicochemical motifs in T cell receptor sequences as a potential biomarker for high-grade serous ovarian carcinoma. PLoS One.

[4] Zhu H, Wei M, Xu J, Hua J, Liang C, Meng Q, Zhang Y, Liu J, Zhang B, Yu X, Shi S (2020). PARP inhibitors in pancreatic cancer: Molecular mechanisms and clinical applications. Mol Cancer.

[5] Kaur K, Rajeshwari M, Gurung N, Kumar H, Sharma M C, Yadav R, Kumar S, Manchanda S, Singhal S, Mathur S R (2020). Uterine tumor resembling ovarian sex cord tumor: A series of six cases displaying varied histopathological patterns and clinical profiles. Indian J Pathol Microbiol.

[6] Chen Z, Guo X, Sun S, Lu C, Wang L (2020). Serum miR-125b levels associated with epithelial ovarian cancer (EOC) development and treatment responses. Bioengineered.

[7] Hurley L C, Levin N K, Chatterjee M, Coles J, Muszkat S, Howarth Z, Dyson G, Tainsky M A (2020). Evaluation of paraneoplastic antigens reveals TRIM21 autoantibodies as biomarker for early detection of ovarian cancer in combination with autoantibodies to NY-ESO-1 and TP53. Cancer Biomark.

[8] Miao R, Badger T C, Groesch K, Diaz-Sylvester P L, Wilson T, Ghareeb A, Martin J A, Cregger M, Welge M, Bushell C, Auvil L, Zhu R, Brard L, Braundmeier-Fleming A (2020). Assessment of peritoneal microbial features and tumor marker levels as potential diagnostic tools for ovarian cancer. PLoS One.

[9] Singh M S, Goldsmith M, Thakur K, Chatterjee S, Landesman-Milo D, Levy T, Kunz-Schughart L A, Barenholz Y, Peer D (2020). An ovarian spheroid based tumor model that represents vascularized tumors and enables the investigation of nanomedicine therapeutics. Nanoscale.

[10] Nomelini R S, Carrijo C A, Abdulmassih F F, da Silva R D, Tavares-Murta B C, Murta E C (2019). Neutrophil-to-lymphocyte ratio and platelet count as prognostic factors in ovarian malignancies. J Cancer Res Ther.

[11] Salminen L, Nadeem N, Jain S, Grènman S, Carpén O, Hietanen S, Oksa S, Lamminmäki U, Pettersson K, Gidwani K, Huhtinen K, Hynninen J (2020). A longitudinal analysis of CA125 glycoforms in the monitoring and follow up of high grade serous ovarian cancer. Gynecol Oncol.

[12] Iwahashi N, Inai Y, Minakata S, Sakurai S, Manabe S, Ito Y, Ino K, Ihara Y (2020). C-Mannosyl tryptophan increases in the plasma of patients with ovarian cancer. Oncol Lett.

[13] Maděrka M, Pilka R, Neubert D, Hambálek J (2019). New serum tumor markers S100, TFF3 and AIF-1 and their possible use in oncogynecology. Ceska Gynekol.

[14] Turashvili G, Fix D J, Soslow R A, Park K J (2020). Wilms Tumor of the Ovary: Review of the Literature and Report of 2 Cases. Int J Gynecol Pathol.

[15] Chin C D, Fares C M, Konecny G E, Rao J (2020). Biomarkers that may predict response to immunotherapy in ovarian malignancies. Curr Opin Obstet Gynecol.

[16] Wolf D, Fiegl H, Zeimet A G, Wieser V, Marth C, Sprung S, Sopper S, Hartmann G, Reimer D, Boesch M (2020). High RIG-I expression in ovarian cancer associates with an immune-escape signature and poor clinical outcome. Int J Cancer.

[17] Sullivan M W, Modesitt S C (2019). When to Worry about Cancer: Concurrent Carcinoma and Recurrence in Borderline Ovarian Tumors. South Med J.

[18] Li H, Terry M B, Antoniou A C, Phillips K A, Kast K, Mooij T M, Engel C, Noguès C, Stoppa-Lyonnet D, Lasset C, Berthet P, Mari V, Caron O, Barrowdale D (2020). Alcohol Consumption, Cigarette Smoking, and Risk of Breast Cancer for BRCA1 and BRCA2 Mutation Carriers: Results from The BRCA1 and BRCA2 Cohort Consortium. Cancer Epidemiol Biomarkers Prev.

[19] Ascierto P A, Bifulco C, Buonaguro L, Emens L A, Ferris R L, Fox B A, Delgoffe G M, Galon J, Gridelli C, Merlano M, Nathan P, Odunsi K, Okada H, Paulos C M, Pignata S (2019). Perspectives in immunotherapy: Meeting report from the 'Immunotherapy Bridge 2018' (28-29 November, 2018, Naples, Italy). Journal for ImmunoTherapy of Cancer.

[20] Zong L, Zhou Y, Zhang M, Chen J, Xiang Y (2020). VISTA expression is associated with a favorable prognosis in patients with high-grade serous ovarian cancer. Cancer Immunol Immunother.

